# Large Language Model Decision Support for Cranial CT in Pediatric Head Trauma

**DOI:** 10.3390/diagnostics16162558

**Published:** 2026-08-14

**Authors:** Ezgi Cesur, Ali Halici, Nursel Kurtoglu

**Affiliations:** 1 Emergency Medicine Department, Kütahya City Hospital, 43020 Kütahya, Türkiye; drezgiyagyemez@gmail.com; 2 Department of Emergency Medicine, Faculty of Medicine, Kütahya Health Sciences University, 43100 Kütahya, Türkiye; nursel.kurtoglu@ksbu.edu.tr

**Keywords:** pediatric head trauma, large language model, clinical decision support, computed tomography, emergency department

## Abstract

**Background**: Pediatric head trauma is a common reason for emergency department presentation. Although most children have minor injuries, a small proportion harbor clinically important traumatic brain injuries requiring urgent intervention. Artificial intelligence (AI) may offer structured support in computed tomography (CT) decision making, but evidence regarding the performance of general-purpose large language models in pediatric head trauma remains limited. Objective: To evaluate the association between AI-based cranial CT recommendations and clinically meaningful outcomes in pediatric patients with blunt head trauma and to assess the diagnostic performance and clinical utility of the model. **Methods**: This retrospective single-center observational study included pediatric patients younger than 18 years with blunt head trauma who underwent cranial CT imaging and had complete outcome data. A general-purpose large language model generated binary CT recommendations (“CT recommended” or “CT not recommended”) using structured clinical information available at the time of emergency department presentation. The primary outcome was a composite adverse clinical outcome defined as the occurrence of at least one of the following: emergency surgical intervention, intensive care unit admission, intubation, neurological sequelae or mortality. Diagnostic performance metrics, calibration analysis and decision curve analysis were performed. **Results**: A total of 819 pediatric patients were included, and the AI model recommended CT in 530 patients (64.7%). The primary outcome occurred in 143 patients (17.5%) and was significantly more frequent in the CT-recommended group than in the CT-not recommended group (24.5% vs. 4.5%; OR 6.90, 95% CI 3.82–12.45; *p* < 0.001). Abnormal CT findings, emergency surgery, intubation and neurological sequelae were also significantly more common in patients for whom CT was recommended by the AI system. For the primary outcome, the AI recommendation demonstrated a sensitivity of 90.9%, specificity of 40.8%, positive predictive value of 24.5% and negative predictive value of 95.5%. Calibration analysis showed acceptable agreement between predicted probabilities and observed event rates. Decision curve analysis demonstrated greater net benefit than both the “treat-all” and “treat-none” strategies across a range of threshold probabilities. **Conclusions**: In this clinically selected cohort of pediatric patients with blunt head trauma who underwent cranial CT imaging, AI-based CT recommendations were strongly associated with adverse clinical outcomes and demonstrated high sensitivity and negative predictive value for identifying children at risk of clinically important events. These findings suggest that, within a clinically selected cohort of children who underwent cranial CT imaging, AI-generated CT recommendations were associated with clinically meaningful outcomes. However, these results should not be interpreted as validation of CT decision making in the broader pediatric head trauma population and require prospective validation in unselected cohorts.

## 1. Introduction

Injuries remain one of the leading causes of morbidity and mortality in children, and head trauma is among the most frequent reasons for presentation to pediatric emergency departments. The vast majority of these patients have minor head injury and recover without clinically significant sequelae [1,2]. However, a small subset may harbor traumatic intracranial injuries that require urgent neurosurgical intervention, intensive care or close neurological observation. Cranial CT plays a central role in the rapid identification of these injuries and remains the most widely used imaging modality in the acute setting. Nevertheless, CT is not necessary for every child with head trauma [3,4,5]. Unnecessary imaging exposes children to ionizing radiation, may increase anxiety for both patients and families and contributes to healthcare costs and resource utilization. Therefore, one of the main challenges in the evaluation of pediatric head trauma is to distinguish children with clinically important traumatic brain injury from the much larger group of patients who are at very low risk and can be managed safely without CT imaging.

In the emergency department, decisions regarding CT imaging in children with head trauma are often made under time pressure and in the setting of substantial diagnostic uncertainty. This challenge may be even more pronounced in busy clinical environments, where high patient volumes, varying levels of physician experience, and concerns about missing a significant injury can influence decision making. In such circumstances, clinicians may benefit from tools that provide structured and consistent support based on the information available at the time of presentation [6,7,8]. Recent advances in artificial intelligence have led to the development of clinical decision-support systems capable of integrating multiple clinical variables and generating real-time recommendations. In emergency medicine, these approaches have shown promise in assisting risk stratification, reducing variability in clinical practice, and supporting more standardized decision making. In the context of pediatric head trauma, such tools may help clinicians identify low-risk patients more confidently while maintaining attention to those who may require urgent evaluation and treatment [9,10]. Several validated clinical decision rules, including PECARN, CATCH and CHALICE, have been developed to support CT decision making in pediatric head trauma. These rules prioritize high sensitivity for clinically important traumatic brain injury and remain widely used in clinical practice. Unlike rule-based approaches, large language models generate recommendations by integrating multiple clinical variables without relying on predefined decision algorithms.

Despite this growing interest, evidence regarding the use of general-purpose large language models in pediatric head trauma remains limited. Most published studies have focused on the detection of intracranial hemorrhage on imaging or on the performance of machine learning models developed for specific datasets. Relatively few studies have examined whether recommendations generated from structured clinical information are associated with outcomes that are directly relevant to patient care, such as the need for neurosurgical intervention, intensive care admission, intubation, neurological sequelae or mortality [11,12,13]. Assessing these outcomes is important because the clinical value of a decision-support system depends not only on its ability to generate plausible recommendations but also on whether those recommendations meaningfully reflect the risk of adverse outcomes.

The aim of this study was to evaluate the performance of a general-purpose large language model in generating cranial CT recommendations for pediatric patients with blunt head trauma using structured clinical information available at the time of emergency department presentation. To ensure reliable outcome ascertainment, the analysis was limited to children who underwent cranial CT imaging and for whom complete clinical outcome data were available. Within this clinically selected cohort, we examined whether AI-based CT recommendations were associated with meaningful outcomes, including adverse clinical events, abnormal CT findings, emergency surgical intervention, and intubation requirement. In addition, we assessed the diagnostic performance and potential clinical utility of the model using calibration and decision curve analyses. We hypothesized that AI-generated recommendations would identify patients with a higher risk profile and could serve as a supportive tool in the evaluation of pediatric head trauma, particularly by helping clinicians more confidently recognize children at low risk for clinically important injury.

## 2. Materials and Methods

### 2.1. Study Design and Setting

This retrospective, single-center observational implementation study was conducted in the emergency department of a tertiary university hospital. Pediatric patients presenting with head trauma during the predefined study period were screened through the institutional electronic medical record system. The study design, data collection process, and reporting strategy were developed in accordance with the STROBE recommendations for observational studies.

### 2.2. Study Population

Patients younger than 18 years of age who were evaluated for blunt head trauma in the emergency department, had sufficiently documented clinical data at presentation, and underwent cranial computed tomography imaging were included in the study. Patients with penetrating head trauma, duplicate visits, transferred patients with insufficient initial assessment data, cases with missing key variables, and records lacking outcome information were excluded. In patients with multiple admissions, only the first visit was included in the analysis.

During the study period, 1576 pediatric patients presented to the emergency department with head trauma. Of these, 647 patients who did not undergo cranial CT imaging were excluded. Additional exclusions included missing key clinical variables required for AI assessment (*n* = 53), missing outcome data (*n* = 9), duplicate encounters (*n* = 16), penetrating head trauma (*n* = 5) and transferred patients with incomplete initial assessment data (*n* = 27). After applying the eligibility criteria, 819 patients who underwent cranial CT imaging and had complete clinical and outcome data were included in the final analysis.

Because cranial CT imaging is not routinely performed in all pediatric head trauma presentations, the study cohort consisted only of patients for whom imaging was clinically obtained after physician evaluation. CT decisions were made according to clinical findings at presentation, trauma mechanism, symptoms, physical examination findings, and the responsible physician’s clinical judgment. Accordingly, the present study was not designed as a universal screening validation study for all pediatric head trauma presentations but, rather, as an implementation and agreement analysis within a clinically selected CT-imaged cohort.

This approach allowed more reliable verification of intracranial pathology and more accurate classification of clinical outcomes through the availability of imaging findings. Nevertheless, the results should not be directly generalized to patients considered very low risk and discharged without imaging.

### 2.3. Data Sources and Data Collection

Demographic, clinical, imaging and outcome data were obtained from electronic medical records using a standardized data collection form. Recorded variables included age, sex, trauma mechanism, trauma severity, vomiting, seizure, loss of consciousness, neurological deficit, hospital admission, intensive care unit requirement, intubation, surgical intervention, imaging findings, neurological sequelae and mortality.

To improve clinical relevance, trauma mechanisms were categorized as low-, moderate-, or high-energy injuries according to predefined criteria. Low-energy trauma included same-level falls and minor household impacts. Moderate-energy trauma included bicycle-related injuries, falls down stairs, and falls from a height of less than 1 m. High-energy trauma included motor vehicle collisions, pedestrian vehicle accidents, falls from a height greater than 3 m and crush injuries.

Only findings explicitly documented in the medical record were included in the database. Ambiguous, conflicting or retrospectively added information was not used. Cases with missing key variables required for AI evaluation or outcome assessment were excluded, and all analyses were performed using a complete-case approach.

### 2.4. Large Language Model Assessment

A large language model was used to generate advisory recommendations regarding the need for cranial CT in pediatric patients presenting with blunt head trauma. Assessments were performed using ChatGPT 5.2 (OpenAI, San Francisco, CA, USA). Case evaluations were performed between January and February 2026 using the same model version. No study-specific model training, fine-tuning, or parameter updating was performed in this study. ChatGPT 5.2 was used as a pretrained, general-purpose large language model, and the study dataset was used exclusively for retrospective assessment of model-generated recommendations rather than for model training.

A standardized prompt was developed and applied uniformly to all study cases to ensure methodological consistency. The clinical variables incorporated into the prompt were prespecified before the AI assessment and were selected based on their clinical relevance to pediatric head trauma evaluation and CT decision making. For each patient, predefined clinical variables collected during the initial emergency department assessment were entered into the prompt template. These variables included demographic characteristics (age and sex), mechanism and severity of trauma, Glasgow Coma Scale score and relevant clinical findings, including loss of consciousness, nausea/vomiting, headache, seizure, lethargy, abnormal behavior, pupillary abnormalities, focal neurological deficits, non-frontal scalp hematoma, suspicion of basilar skull fracture, palpable or depressed skull fracture and clinical deterioration during the observation period.

The model was explicitly instructed to evaluate each patient independently using only information available at the time of the initial emergency department presentation. It was instructed not to infer or assume missing clinical information and not to use radiological findings, hospital course, follow-up information, final diagnosis, treatment, neurological outcomes, mortality or any other post-presentation data. The prompt further emphasized that the generated recommendation was intended solely as a decision-support output and not as a substitute for physician judgment.

For each case, the model was required to generate only one of two predefined binary outputs, “CT recommended” or “CT not recommended”, without providing explanations, reasoning, confidence estimates or additional text. The same prompt structure and output constraints were applied to all patients throughout the study.

Each patient was evaluated independently using the same standardized prompt format. To minimize contextual carry-over between cases, separate chat sessions were used for individual evaluations. Because no study-specific model training or parameter estimation was performed, conventional internal model validation was not applicable. Formal test–retest assessment and inter-run agreement analyses were not performed and are acknowledged as limitations of the present study.

#### Exploratory PECARN Analysis

To provide clinical context, PECARN risk classification was retrospectively reconstructed using the clinical variables available at the initial emergency department assessment. Patients were initially categorized according to PECARN criteria, and for binary diagnostic performance analyses, intermediate- and high-risk categories were combined and considered PECARN-positive, whereas low-risk patients were considered PECARN-negative. Diagnostic performance measures for PECARN were calculated for the primary adverse clinical outcome and descriptively compared with those of the LLM recommendation. This exploratory analysis was not intended as a formal superiority comparison between the two approaches.

### 2.5. Reference Standard and Outcomes

The primary outcome was predefined as an adverse clinical outcome, defined as the presence of at least one clinically important event related to head trauma. These events included surgical intervention, intensive care unit admission, intubation, neurological sequelae or mortality.

Secondary outcomes included abnormal CT findings, hospital admission, intensive care requirement, emergency department intubation, surgical intervention, neurological sequelae and mortality.

Because cranial CT imaging is not routinely performed in all pediatric head trauma patients, the primary outcome was based on clinically relevant adverse outcomes rather than imaging findings alone in order to reduce potential verification bias associated with imaging-only analyses.

### 2.6. Measures to Reduce Bias

Several measures were taken to reduce potential bias. First, only variables available at the time of presentation were used for AI evaluation, and no subsequent clinical information was provided to the model. Second, all cases were assessed using the same standardized prompt template. Third, clinical outcomes were obtained independently from source records without reference to AI responses. Fourth, predefined subgroup analyses were performed according to clinically relevant categories such as age groups and trauma severity. Finally, the potential clinical utility of the AI approach was evaluated not only through diagnostic performance metrics but also through decision curve analysis.

### 2.7. Ethics Approval

This study was approved by the Kütahya Health Sciences University Non-Interventional Clinical Research Ethics Committee (Approval No. 2025/11-02; 15 September 2025). The requirement for informed consent was waived because of the retrospective observational design of the study. The study was conducted in accordance with the principles of the Declaration of Helsinki.

### 2.8. Statistical Analysis

Continuous variables were summarized as mean ± standard deviation or median (IQR), depending on distribution characteristics. Categorical variables were presented as counts and percentages. Comparisons between groups were performed using Student’s *t*-test or Mann–Whitney U test for continuous variables and chi-square test or Fisher’s exact test for categorical variables.

The diagnostic performance of the AI-based CT recommendation and the exploratory PECARN classification was evaluated for the primary adverse clinical outcome using sensitivity, specificity, positive predictive value, negative predictive value, positive likelihood ratio, and negative likelihood ratio.

Model performance was additionally evaluated in terms of calibration and clinical utility. Calibration analyses were performed exclusively for the multivariable logistic model derived from AI recommendations and clinical covariates. Because the standalone AI output was binary, conventional calibration assessment was not applicable to the LLM recommendation itself. Agreement between predicted probabilities and observed event rates was assessed using calibration plots.

Clinical utility was evaluated using decision curve analysis across a range of threshold probabilities. The multivariable model was compared with the “treat-all” and “treat-none” strategies. In addition, an exploratory decision curve analysis based solely on the binary AI recommendation was also performed.

Exploratory analyses were additionally conducted for false-negative cases classified as “CT not recommended” despite subsequent adverse clinical outcomes. These cases were evaluated according to age groups and trauma severity subgroups.

All statistical tests were two-sided, and *p* values < 0.05 were considered statistically significant. Analyses were performed using IBM SPSS Statistics softwarefor Windows, version 27.0 (IBM Corp., Armonk, NY, USA), while additional graphical analyses were conducted using appropriate statistical software.

## 3. Result

### 3.1. Study Population and Baseline Characteristics

A total of 819 pediatric patients with blunt head trauma who underwent cranial CT imaging were included. The mean age was 8.42 ± 4.48 years, and 413 patients (50.4%) were female. Age and sex distributions were similar between groups (*p* = 0.155 and *p* = 0.325, respectively). Demographic and baseline clinical characteristics of the study population are presented in Table 1.

Patients for whom CT was recommended by the AI model more frequently had moderate- or high-energy trauma, loss of consciousness, vomiting, headache, seizure and neurological deficits (all *p* < 0.001). Suspicion of basilar skull fracture and palpable depressed fracture were also more common in this group (*p* = 0.017 and *p* < 0.001, respectively).

### 3.2. Clinical Outcomes

Adverse clinical outcomes were observed in 143 patients (17.5%) and were more frequent in the CT-recommended group (OR: 6.90, 95% CI: 3.82–12.45). Abnormal CT findings (OR: 6.58, 95% CI: 3.71–11.66), emergency surgery (OR: 5.79, 95% CI: 2.75–12.18), ED intubation (OR: 6.15, 95% CI: 2.93–12.93) and neurological sequelae (OR: 6.45, 95% CI: 2.29–18.14) were all more frequent in the CT-recommended group (all *p* < 0.001). Mortality did not differ significantly between groups (OR: 3.04, 95% CI: 0.67–13.82; *p* = 0.130). Clinical outcomes according to AI-based CT recommendation are presented in Table 2.

Among the 289 patients for whom CT was not recommended by the AI model, 13 (4.5%) experienced the primary adverse clinical outcome. Review of these false-negative cases showed that several had clinically concerning features, including high-energy trauma (*n* = 2), Glasgow Coma Scale score < 14 (*n* = 3), seizure (*n* = 1), loss of consciousness (*n* = 1), vomiting (*n* = 5), headache (*n* = 6), neurological deficits (*n* = 3) and palpable depressed skull fracture (*n* = 2).

### 3.3. Clinical Outcomes According to PECARN Classification

As an exploratory contextual analysis, PECARN classification was retrospectively applied to the same cohort. For binary analyses, intermediate- and high-risk categories were combined and considered PECARN-positive. Adverse clinical outcomes were more frequent among PECARN-positive patients than PECARN-negative patients (OR: 2.56, 95% CI: 1.34–4.89; *p* = 0.003). Similarly, abnormal CT findings (OR: 2.40, 95% CI: 1.29–4.47; *p* = 0.005), emergency surgery (OR: 2.60, 95% CI: 1.11–6.10; *p* = 0.023) and ED intubation (OR: 2.75, 95% CI: 1.18–6.45; *p* = 0.015) were more frequent among PECARN-positive patients. Neurological sequelae were numerically more frequent among PECARN-positive patients, although the difference did not reach statistical significance (*p* = 0.060). Mortality was observed only in PECARN-positive patients, but the difference was not statistically significant (*p* = 0.240).

### 3.4. Diagnostic Performance

The diagnostic performance of the LLM-based CT recommendation and PECARN classification for the primary adverse clinical outcome is presented in Table 3. Both approaches demonstrated high sensitivity, with PECARN showing slightly higher sensitivity than the LLM-based recommendation (92.3% vs. 90.9%). However, the LLM-based recommendation showed substantially higher specificity (40.8% vs. 17.6%).

### 3.5. Calibration of the Multivariable Prediction Model

Calibration analysis of the multivariable model showed good agreement between predicted probabilities and observed event rates across risk groups. The model demonstrated consistent calibration in both low- and high-risk ranges, with minor deviations in intermediate risk levels.

**Figure 1:**
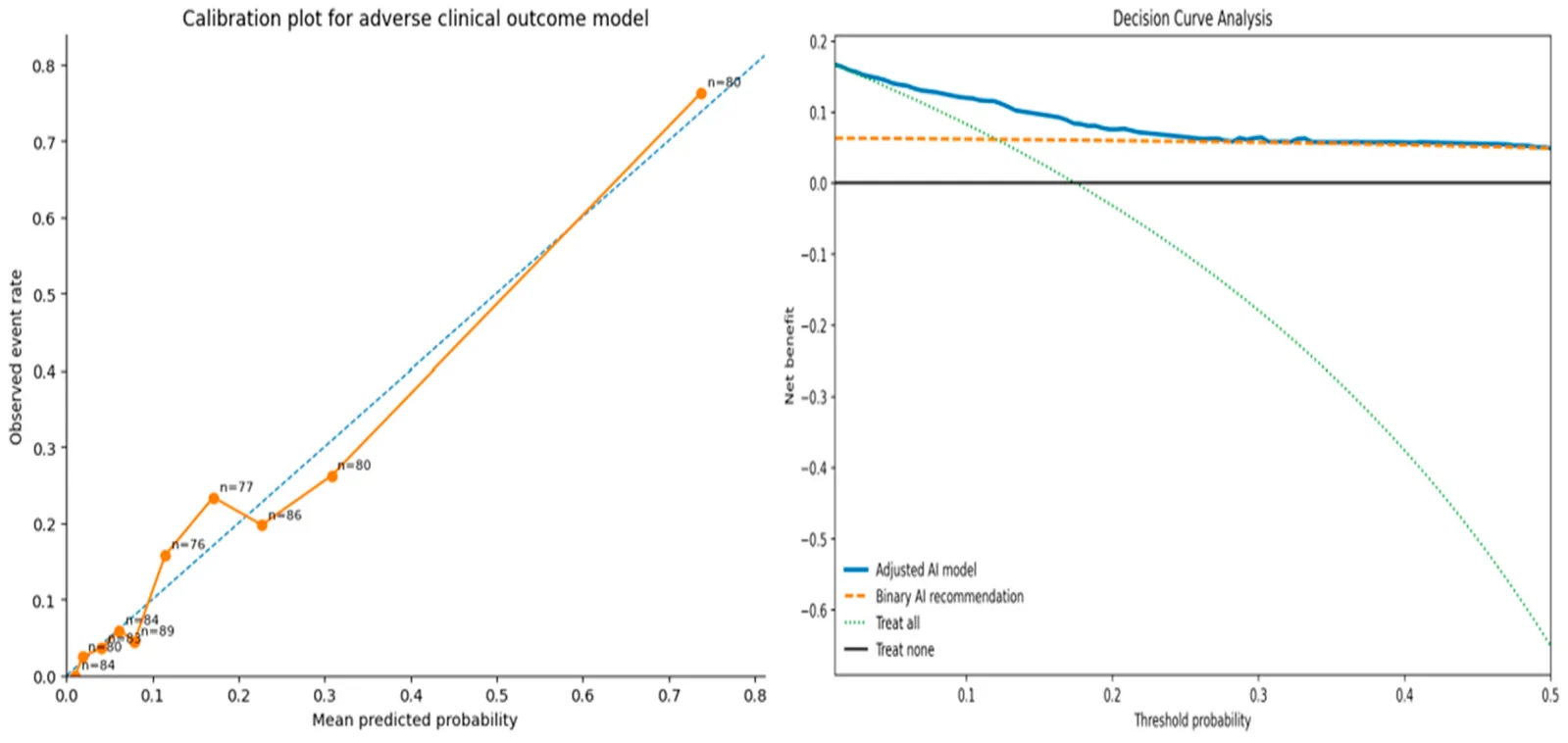
**Calibration plot and decision curve analysis of the AI model for adverse clinical outcomes.** The calibration plot shows the observed event rates against the mean predicted probabilities; the orange line represents the observed event rates, while the blue dashed diagonal line represents ideal calibration. In the decision curve analysis, the blue solid line represents the adjusted AI model, the orange dashed line represents the binary AI recommendation, the green dotted line represents the “treat-all” strategy, and the black solid line represents the “treat-none” strategy.

Decision curve analysis showed that the multivariable model provided higher net benefit than both the “treat-all” and “treat-none” strategies across a range of threshold probabilities. The binary AI-based CT recommendation also demonstrated net benefit, although it remained lower than that of the multivariable model, with net benefit being more pronounced in the low- to intermediate-threshold probability range. These findings are illustrated in Figure 1.

## 4. Discussion

In this study, AI-based CT recommendation was found to be associated with clinically meaningful outcomes in pediatric patients with head trauma who underwent cranial CT imaging. This finding suggests that the model tends to distinguish patients with a higher risk profile, was associated with clinically meaningful outcomes within a clinically selected CT imaged cohort and demonstrated agreement with established clinical risk indicators.

In our cohort, patients for whom CT was recommended by the AI system demonstrated a higher risk profile in terms of traumatic brain injury-related clinical symptoms, physical examination findings and trauma characteristics. Given that the model was evaluated using structured clinical information commonly considered during pediatric head trauma assessment, this finding suggests that the AI recommendations were generally consistent with established clinical risk indicators rather than randomly generated decisions. It has long been recognized that variables such as trauma mechanism, neurological findings, vomiting and loss of consciousness play an important role in predicting clinically important traumatic brain injury in children [1,14]. In recent years, several studies have also shown that AI-based approaches may support clinical decision making by simultaneously evaluating multiple clinical inputs [12,15,16]. In this context, our findings suggest that, when provided with clinically meaningful patient data, the AI model was able to generate CT recommendations broadly compatible with current clinical assessment approaches.

Patients for whom CT was recommended by the AI system had higher rates of adverse clinical outcomes, abnormal CT findings, emergency surgical intervention and intubation requirement, indicating that the model output was aligned with clinically meaningful outcomes. In contrast, no significant difference was observed between groups in terms of mortality. However, the low mortality rate and limited number of events may have reduced the ability to demonstrate statistical significance for this outcome. Taken together, these findings suggest that the AI recommendation was not merely reflecting symptoms or imaging decisions but was also capable of distinguishing patients at risk for clinically important outcomes. It is well established that clinically important traumatic brain injury in pediatric head trauma is relatively uncommon but associated with serious consequences, making accurate risk stratification critically important. Recent studies evaluating AI-based models have similarly demonstrated that predictions generated through combined clinical and demographic variables may correlate with clinically relevant outcomes [17,18]. From this perspective, our findings support the notion that AI-based decision-support approaches may provide not only theoretical but also practical clinical value.

When diagnostic performance was evaluated, the AI-based CT recommendation demonstrated high sensitivity and negative predictive value. Sensitivity exceeded 90% for the primary adverse clinical outcome, suggesting a low likelihood of missing patients with clinically important events. Although specificity remained lower than sensitivity, the LLM recommendation demonstrated higher specificity than the exploratory PECARN classification within the same cohort. However, established pediatric head trauma decision rules such as PECARN are designed to prioritize sensitivity for clinically important traumatic brain injury, with reported sensitivities of 96–100% and specificities of approximately 50–60% [19,20,21,22]. Validation studies reporting no missed serious injuries in low-risk groups further support this high-sensitivity approach [23,24]. In our exploratory comparison, PECARN demonstrated slightly higher sensitivity for adverse clinical outcomes, whereas the LLM recommendation showed higher specificity. These differences should not be interpreted as evidence of superiority of either approach, particularly because the comparison was performed for contextual purposes within a CT-enriched cohort and intermediate- and high-risk PECARN categories were combined. The findings therefore suggest that the LLM may have a complementary role rather than serve as a replacement for established decision rules. Unlike PECARN, which applies a predefined and validated decision algorithm, the LLM-based approach was not explicitly instructed to follow a specific clinical decision rule but instead integrated multiple clinical features to generate a CT recommendation. This flexibility may represent a potential advantage of LLM-based decision support; however, its incremental clinical benefit over established decision rules remains to be determined in prospective studies. Importantly, the presence of false-negative cases with clinically important outcomes indicates that the LLM recommendation should not be used as a standalone rule-out tool and should remain an adjunct to physician judgment.

Decision curve analysis suggested that AI-based CT recommendation may provide potential benefit in clinical decision making. The model demonstrated greater net benefit than both the “treat-all” and “treat-none” strategies, particularly in cases where uncertainty existed regarding the necessity of CT imaging. This finding suggests that AI support may help improve the balance between unnecessary imaging and the risk of missing clinically important injuries. Similarly, studies evaluating AI-assisted decision systems in pediatric head trauma have emphasized that these models should be assessed not only in terms of diagnostic accuracy but also regarding their impact on clinical decision making. In particular, AI-assisted approaches have been reported to reduce unnecessary CT utilization while identifying certain traumatic intracranial hemorrhages that might otherwise have been missed [25,26]. Nevertheless, the relatively limited net benefit observed in our study suggests that the model may be more appropriately considered as a supportive tool rather than a standalone decision-making system.

Calibration analysis of the multivariable prediction model demonstrated acceptable agreement between predicted and observed event rates. In other words, adverse clinical outcomes were more frequently observed in patient groups predicted to be at higher risk, whereas lower event rates were observed among those categorized as low risk. The fluctuations observed in some intermediate-risk groups may be related to the limited sample size and uneven distribution of events. Nonetheless, the findings suggest that the AI-based approach did not produce a completely inconsistent or random risk distribution and that there was a generally acceptable relationship between predicted risk levels and observed outcomes. Previous studies have similarly emphasized that AI-based clinical decision-support systems should be evaluated not only by their discriminative ability but also by their clinical reliability and compatibility with real-world decision-making processes [8]. Taken together, these findings suggest that AI-assisted CT recommendation may serve as a useful adjunct in pediatric head trauma evaluation, particularly in the assessment of low-risk patients.

Within this clinically selected cohort of pediatric patients who underwent cranial CT imaging, AI-generated CT recommendations were associated with clinically meaningful outcomes and demonstrated high sensitivity and negative predictive value for adverse clinical events. However, because the study population was restricted to patients who had already undergone CT imaging, these findings should not be interpreted as evidence of performance in the broader pediatric head trauma population or as validation of CT decision making in unselected patients. Rather, the results suggest that, when provided with structured clinical information available at presentation, a general-purpose large language model can identify patients with a higher-risk clinical profile within an already-imaged cohort. Nevertheless, our findings suggest that AI models may be more appropriately considered as supportive tools integrated into clinical assessment rather than independent standalone decision makers [27]. In this context, the present findings should be considered hypothesis-generating rather than practice-changing. Because this was a retrospective single-center study conducted in a clinically selected cohort, the findings should be interpreted with caution and should not be generalized to other clinical settings.

These findings should be interpreted in the context of the emerging literature on large language models in head trauma. Simper et al. showed that an LLM-based model could accurately apply the PECARN algorithm using information extracted from emergency department records, with performance approaching that of pediatric emergency physicians [28]. More recently, Lampros et al. reported that ChatGPT-5.1 generated CT recommendations comparable to the Canadian CT head rule in adults with mild traumatic brain injury [29]. Unlike these studies, which evaluated LLMs alongside established clinical decision rules, our study assessed the performance of a general-purpose LLM without explicitly instructing it to apply a predefined algorithm. Together, these studies suggest that LLMs may support clinical decision making through different approaches, while our findings add evidence for their potential role in pediatric head trauma. Prospective multicenter studies with external validation are therefore needed to confirm the reproducibility and generalizability of these findings and to evaluate the clinical utility of LLM-based decision support in real-time practice, including its impact on CT utilization and clinical decision making.

### 4.1. Limitations

This study has several limitations. First, the retrospective and single-center design may limit the generalizability of the findings, as patient populations and clinical practices may vary across institutions. Differences in patient characteristics, trauma mechanisms, clinical assessment practices, and thresholds for cranial CT imaging may also influence the performance of the LLM in other clinical settings. In addition, no external validation cohort was available. Therefore, the observed performance characteristics should be interpreted as preliminary findings from a single-center dataset and require confirmation in independent populations before broader generalization. Furthermore, the AI model was evaluated retrospectively rather than in a real-time clinical setting; therefore, its direct impact on clinical workflow, physician behavior or decision-making time could not be assessed. Finally, only patients who underwent cranial CT imaging were included, which may have excluded lower-risk patients who did not require imaging. Consequently, the findings should be interpreted as reflecting a selected cohort of patients for whom CT imaging was clinically indicated rather than the entire pediatric head trauma population.

### 4.2. Strengths

Despite these limitations, our study also has several important strengths. First, the study evaluated AI-based CT recommendation not only in relation to imaging findings but also in association with clinically meaningful outcomes such as adverse clinical events, emergency surgical intervention, intubation requirement and intensive care admission. This approach is important because it reflects not only the theoretical diagnostic performance of the AI recommendation but also its relationship with clinically relevant patient outcomes. In addition, the high sensitivity and high negative predictive value observed in our study suggest that the AI-assisted approach may serve as a safe supportive tool, particularly in the evaluation of low-risk patients. Furthermore, the study allowed assessment of the AI approach not only in terms of discriminative performance but also regarding clinical utility and prediction reliability. Evaluating the potential role of AI-based decision-support systems in real-world clinical practice within a patient population such as pediatric head trauma patients, where event rates are relatively low but clinical consequences may be severe, represents another notable strength of this study. We believe that these findings may provide a basis for future prospective multicenter investigations.

## Figures and Tables

**Table 1:** Baseline characteristics by AI-based CT recommendation.

	Total (*n* = 819)	No CT Recommended (*n* = 289)	CT Recommended (*n* = 530)	*p* Value
**Demographic characteristics**				
Age, years	8.42 ± 4.48	8.12 ± 4.68	8.58 ± 4.36	0.155
Female, *n* (%)	413 (50.4)	139 (48.1)	274 (51.7)	0.325
**Trauma characteristics**				
Low-energy trauma, *n* (%)	396 (48.4)	164 (56.7)	232 (43.8)	<0.001
Moderate-energy trauma, *n* (%)	349 (42.6)	113 (39.1)	236 (44.5)	
High-energy trauma, *n* (%)	74 (9.0)	12 (4.2)	62 (11.7)	
**Clinical symptoms and neurological findings**				
Loss of consciousness, *n* (%)	36 (4.4)	1 (0.3)	35 (6.6)	<0.001
Vomiting, *n* (%)	380 (46.4)	109 (37.7)	271 (51.1)	<0.001
Headache, *n* (%)	548 (66.9)	159 (55.0)	389 (73.4)	<0.001
Seizure, *n* (%)	27 (3.3)	1 (0.3)	26 (4.9)	<0.001
Neurological deficit, *n* (%)	36 (4.4)	3 (1.0)	33 (6.2)	<0.001
**Physical examination and skull findings**				
Non-frontal hematoma, *n* (%)	139 (17.0)	43 (14.9)	96 (18.1)	0.239
Basilar skull fracture suspicion *n* (%)	10 (1.2)	0 (0.0)	10 (1.9)	0.017 *
Palpable depressed fracture, *n* (%)	29 (3.5)	2 (0.7)	27 (5.1)	<0.001
Clinical deterioration during observation, *n* (%)	19 (2.3)	0 (0.0)	19 (3.6)	0.001 *

* Fisher’s exact test used due to sparse expected cell counts.

**Table 2:** Clinical outcomes by AI-based CT recommendation.

Outcome	No CT Recommended (*n* = 289)	CT Recommended (*n* = 530)	OR (95% CI)	*p* Value
Adverse clinical outcome, *n* (%)	13 (4.5%)	130 (24.5%)	6.90 (3.82–12.45)	<0.001
Abnormal CT finding, *n* (%)	14 (4.8%)	133 (25.1%)	6.58 (3.71–11.66)	<0.001
Neurological sequelae, *n* (%)	4 (1.4%)	44 (8.3%)	6.45 (2.29–18.14)	<0.001
ED intubation, *n* (%)	8 (2.8%)	79 (14.9%)	6.15 (2.93–12.93)	<0.001
Emergency surgery, *n* (%)	8 (2.8%)	75 (14.2%)	5.79 (2.75–12.18)	<0.001
Mortality, *n* (%)	2 (0.7%)	11 (2.1%)	3.04 (0.67–13.82)	0.130

**Table 3:** Comparative performance of the LLM recommendation and PECARN.

Approach	Sensitivity (95% CI)	Specificity (95% CI)	PPV (95% CI)	NPV (95% CI)	LR+ (95% CI)	LR− (95% CI)
LLM-based CT recommendation	90.9 (85.1–94.6)	40.8 (37.2–44.6)	24.5 (21.1–28.4)	95.5 (92.5–97.4)	1.54 (1.42–1.67)	0.22 (0.13–0.38)
PECARN classification	92.3 (86.8–95.7)	17.6 (14.9–20.7)	19.2 (16.4–22.3)	91.5 (85.5–95.2)	1.12 (1.06–1.19)	0.44 (0.24–0.79)

## Data Availability

The data presented in this study are available on request from the corresponding author due to privacy and ethical restrictions related to patient confidentiality.
